# The number of brain metastases predicts the survival of non‐small cell lung cancer patients with EGFR mutation status

**DOI:** 10.1002/cnr2.1550

**Published:** 2021-11-12

**Authors:** Jun Shao, Jingwei Li, Lujia Song, Qiuyao He, Yuxuan Wu, Linhui Li, Dan Liu, Chengdi Wang, Weimin Li

**Affiliations:** ^1^ Department of Respiratory and Critical Care Medicine, Frontiers Science Center for Disease‐related Molecular Network, West China Hospital, West China Medical School Sichuan University Chengdu Sichuan China

**Keywords:** EGFR mutation, lung cancer, number of brain metastases, survival

## Abstract

**Background:**

Lung cancer is the common cause of cancer‐related deaths throughout the world, and brain is a frequent metastatic site of lung cancer.

**Aim:**

This research sought to evaluate the impact of the number of brain metastases in prognosticating non‐small cell lung cancer (NSCLC) patients accounting to the role of epidermal growth factor receptor (EGFR) mutations.

**Methods and Results:**

NSCLC patients with brain metastases diagnosed/treated in West China Hospital, Sichuan University between 2009 and 2017 were identified retrospectively. Kaplan–Meier approach was adopted to estimate OS. And we performed univariate and multivariate Cox proportional hazards regression analyses of characteristics related to overall survival (OS) in both EGFR‐mutated and wild‐type cohorts. In total, this study included 611 eligible NSCLC patients with brain metastases. Extracranial metastases and chemotherapy were independent prognostic factors of OS in both cohorts. As the disease progressed, EGFR‐mutated patients had brain metastasis significantly earlier (*P* < .0001), but they also had notably better survival outcomes than wild‐type patients (*P* < .0001). And the number of brain metastases impacted the survival incidence in the progression significantly in both EGFR‐mutated and wild‐type groups (*P* = .0087/.037, respectively).

**Conclusion:**

The number of brain metastases was a prognostic factor for lung cancer patients either with EGFR mutations or with wild‐type EGFR, with larger number indicating more unfavorble clinical outcomes. Patients with EGFR mutations had a better survival.

## 1 INTRODUCTION

Patients of lung cancer are confronted with a poor prognosis, with a 5‐year overall survival less than 20% in most countries.^1^ In 2018, 1 761 000 deaths were attributed to lung cancer throughout the world, among which 690 000 deaths were in China.^2^ Non‐small cell lung cancer (NSCLC) cases constitute 85% of all lung cancer cases and thus most studies to date have focused on this group.^3^ According to the eighth edition of Cancer Staging Manual proposed by the American Joint Committee (AJCC),^4^ the most common pathologic staging for NSCLC is the tumor‐node‐metastases (TNM) staging strategy, considering mainly tumor size, tumor invasiveness in the regional nodes, and extra‐thoracic metastases. With such multifarious objectives, the TNM staging is a critical tool in the diagnosis and treatment of NSCLC patients.

Brain is a frequent metastatic site of primary tumors. Brain metastasis was reported to influence around 20%–40% of cancer patients.^5^ Lung cancer was also reported as the most frequent primary site of brain metastases in solid tumors, responsible for approximately 60% of brain metastases in all cases.^6^ The incidence of brain metastasis of NSCLC patients was suggested to be over 20% at diagnosis, and as the disease progresses, a significant percentage of patients would further develop brain metastasis.^7^ Around 54% of NSCLC patients might be diagnosed with metastasis in central nervous system during their disease course.^8^ As was validated, brain metastasis was correlated with an unfavorable quality of life and a poor prognosis,^9^ implying that the tumor was invasive and the disease deteriorated into a later period. With the presence of brain metastasis, lung cancer patients would have a median overall survival of 4–11 weeks if untreated and of 4–15 months if treated.^10^

The Iressa Pan‐Asia Study (IPASS) established the crucial role of epidermal growth factor receptor (EGFR) in the progression of lung cancer,^11^ which has initiated a new direction to individualize and design clinical treatment of lung cancer patients. Lung cancer patients with a higher expression of EGFR have a higher mortality risk.^8^ And EGFR is easily mutated in lung cancer patients. Roughly 20% of lung adenocarcinoma patients in western countries carry genetic alterations in EGFR, while 40%–60% of patients in East Asia are EGFR mutation positive.^12^ EGFR mutations are suggested to have a significant association with brain metastasis and overall survival. A cumulative increase in the incidence rate of brain metastasis over time was reported in EGFR‐mutated patients.^13^ Despite of that, patients with EGFR alternations showed a markedly better survival in a multi‐institutional research covering 2186 NSCLC patients.^14^

As the incidence of brain metastasis is correlated with EGFR mutation status,^15^ there is debate over whether the number of brain metastases affects survival of NSCLC patients accounting for EGFR mutation status. With the adjustment of EGFR mutation status, the number of brain metastases was suggested to remain a prognostic factor for all patients, but no statistically significance was found.^14^ Further, Balasubramanian and colleagues. reported that the number of brain metastases merely influenced survival outcomes in the wild‐type NSCLC group but had no impact for EGFR‐mutated patients.^16^ However, there still is a paucity of literature regarding this issue.

This research sought to evaluate the impact of the number of brain metastases in prognosticating NSCLC patients accounting for the role of EGFR mutations. We conducted the investigation on the number of brain metastases from various perspectives including Cox proportional hazards regression analyses and time trends illustration. Also, we added to the real‐world evidence of EGFR mutations influencing the survival outcomes in NSCLC patients with brain metastases. And we innovatively took the different subtypes of EGFR mutations into consideration.

## 2 PATIENTS AND METHODS

### 2.1 Study population

NSCLC patients with at least one brain metastasis, known EGFR mutation status, and complete follow‐up information who were diagnosed/treated in West China Hospital between 2009 and 2017 were identified and included in our study. Before enrollment, patient informed consent was achieved. This research was conducted with the approval of the Institutional Review Board in West China Hospital, Sichuan University (NO 2018.420), and confidentiality was ensured throughout our research.

### 2.2 Characteristics

Demographical and clinical characteristics were downloaded from the Lung Cancer Database of West China Hospital, Sichuan University, including patient age, sex, smoking status, carcinoembryonic antigen (CEA) level, histology subtypes, pathologic TNM stage of the primary lung tumor, EGFR mutation details, presence of extracranial metastases, metastatic sites, exact number of brain metastases, and treatment history. In our study, all patients underwent real‐time PCR to evaluate their EGFR mutation status (including mutations, deletions and insertions). Exon 19 deletion, L858R, G719X, and L861Q were the main mutations we focused on, with the rest of subtypes labeled together as “other.” Erlotinib, gefitinib, and afatinib were the mainly considered TKIs, used commonly in patients with EGFR mutations. Histology subtypes were based on pathological or cytologic results, classified into lung adenocarcinoma (LUAD), lung squamous carcinoma (LUSC), lung adenosquamous carcinoma (LASC), and others. As an indicator, brain imaging can clearly show the occurrence of brain metastasis and calculate the exact number of brain metastases.

New incidence of brain metastasis was calculated from the initial diagnosis of lung cancer for each individual subjects. The outcome variable, overall survival (OS), was measured from the initial time of brain metastasis to the day of reported death due to any cause according to follow‐ups. If the exact date of death was not known, the date of the patient's last available follow‐up would be used for further survival analyses.

### 2.3 Data examination

Data retrieved directly from the online database were to be examined by two authors independently. Mistakes, outliers, or any other inconsistent information were corrected before univariate analysis. Any disagreement was carefully dealt with and finally resolved by a consensus.

### 2.4 Statistical analyses

Kaplan–Meier approach was adopted to estimate OS. And we further performed univariate and multivariate Cox proportional hazards regression analyses of characteristics related to OS in both EGFR‐mutated and wild‐type cohorts separately. Two‐tailed *P*‐values less than .05 were deemed as significant. All analyses in our research were completed using R version 3.6.0.

Variables entering multivariate analysis were the ones in a potentially significant relationship with OS, carefully chosen according to their results in univariate analysis. And to avoid missing any clinically important variables, we loosened the criteria to include variables with *P*‐values less than .1 in our multivariate analysis.

## 3 RESULTS

### 3.1 Univariate and multivariate analyses

A total of 611 NSCLC patients who harbored information on EGFR mutation status (304 EGFR‐mutated patients and 307 EGFR wild‐type patients) and developed brain metastasis in their disease progression were identified for the present analysis. As disease progressed, all patients included in our research developed metastatic lesions in brains. The results of our univariate and multivariate analyses to recognize possible risk factors of OS are shown in Table 1 and a comparison on the influential factors between the EGFR‐mutated cohort and the wild‐type cohort is also exhibited.

**TABLE 1:** Univariate and multivariate Cox proportional hazards regression analysis of characteristics related with overall survival

Characteristics	EGFR‐mutated cohort (*N* = 304)				EGFR wild‐type cohort (*N* = 307)				
	Univariate analysis		Multivariate analysis		Univariate analysis		Multivariate analysis		
	HR (95% CI)	*P*‐value	HR (95% CI)	*P*‐value	HR (95% CI)	*P*‐value	HR (95% CI)	*P*‐value
Age								
≥60 years	1.290 (0.956–1.741)	0.096			1.198 (0.917–1.565)	0.186		
<60 years	1				1			
Sex *n* (%)								
Male	1.377 (1.025–1.849)	0.034	1.511 (1.119–2.039)	0.007	1.321 (0.981–1.779)	0.067		
Female	1		1		1			
Smoking status								
Smokers	1.116 (0.812–1.535)	0.499			1.390 (1.051–1.839)	0.021	1.338 (1.006–1.779)	0.046
Nonsmokers	1				1		1	
CEA level								
Negative	1				1	0.683		
Positive	1.140 (0.848–1.533)	0.385			1.058 (0.808–1.384)			
Histology								
LUAD	1	0.767			1	0.060		
LUSC	1.364 (0.740–2.515)				1.162 (0.827–1.633)			
LASC	1.224 (0.454–3.302)				1.097 (0.450–2.672)			
Others	1.037 (0.299–2.936)				2.060 (1.212–3.500)			
Extracranial metastases (ECM) *n* (%)								
NO	1	<0.001	1	<0.001	1	<0.001	1	<0.001
YES	2.159 (1.613–2.890)		2.198 (1.634–2.955)		2.381 (1.825–3.106)		2.468 (1.885–3.230)	
No. of brain metastases								
1	1	0.010	1	0.047	1	0.039	1	0.089
2	1.102 (0.680–1.786)		1.075 (0.659–1.754)		1.034 (0.639–1.365)		1.004 (0.649–1.553)	
≥3	1.632 (1.164–2.286)		1.492 (1.061–2.097)		1.261 (0.973–1.634)		1.353 (1.038–2.091)	
EGFR‐mutated types								
Exon 19 deletion	1	0.958						
L858R	1.115 (0.712–1.746)							
G719X	0.780 (0.280–2.177)							
L861Q	0.997 (0.357–2.782)							
Other	0.770 (0.106–5.603)							
First‐line treatment								
SRS ± surgery	1	0.771			1	0.325		
WBRT ± surgery	0.654 (0.246–1.735)				0.612 (0.235–1.594)			
SRS + WBRT	0.819 (0.331–2.027)				0.962 (0.561–1.651)			
Chemotherapy								
YES	1	0.054	1	0.010	1	0.006	1	0.030
NO	1.347 (0.995–1.823)		1.497 (1.100–2.039)		1.628 (1.152–2.300)		1.473 (1.038–2.091)	
TKI‐targeted therapy								
NO	1.123 (0.712–1.772)	0.236	1.085 (0.680–1.733)	0.732				
YES	1		1					

Abbreviations: CEA, carcinoembryonic antigen; ECM, extracranial metastases; EGFR, epidermal growth factor receptor; LASC, lung adenosquamous carcinoma; LUAD, lung adenocarcinoma; LUSC, lung squamous carcinoma; No. of brain metastases, number of brain metastases; SRS, stereotactic radiosurgery; TKI, tyrosine kinase inhibitors; WBRT, whole brain radiation therapy.

On univariate analysis, more brain metastases (*P* = .010 in the EGFR‐mutated group; *P* = .039 in the wild‐type group) and occurrence of extracranial metastases (*P* ≤ .001 in both groups) were suggested to be shared risk factors. And on multivariate analysis, extracranial metastases condition and chemotherapy adoption condition (absence of chemotherapy: HR = 1.497, 95% CI: 1.100–2.039, *P* = .010 in EGFR‐mutated group; HR = 1.473, 95% CI: 1.038–2.091, *P* = 0.030 in wild‐type group) impacted OS in both cohorts independently.

For EGFR‐mutated patients, it turned out that sex (*P* = .007), extracranial metastases condition (*P* < .001), number of brain metastases (*P* = .047), and chemotherapy adoption condition (*P* = .010) were independent prognostic factors. And the impact of TKI‐targeted therapy was insignificant (*P* = .732) in our patients. Surprisingly, the specific EGFR‐mutated subtypes did not make a difference on the overall survival (*P* = .958 in the univariate analysis).

In relation to EGFR wild‐type patients, In the multivariate analysis, ever‐smoking (HR = 1.338, 95% CI = 1.006–1.779, *P* = .046), the occurrence of extracranial metastases (HR = 2.468, 95% CI = 1.885–3.230, *P* < .001), lack of chemotherapy (HR = 1.473, 95% CI = 1.038–2.091, *P* = .030) were predictors of undesired OS independently. The number of brain metastases narrowly had a statistically significant impact on the overall survival of wild‐type patients (*P* = .089).

### 3.2 Impact of EGFR mutation status on brain metastasis incidence and survival

**FIGURE 1:**
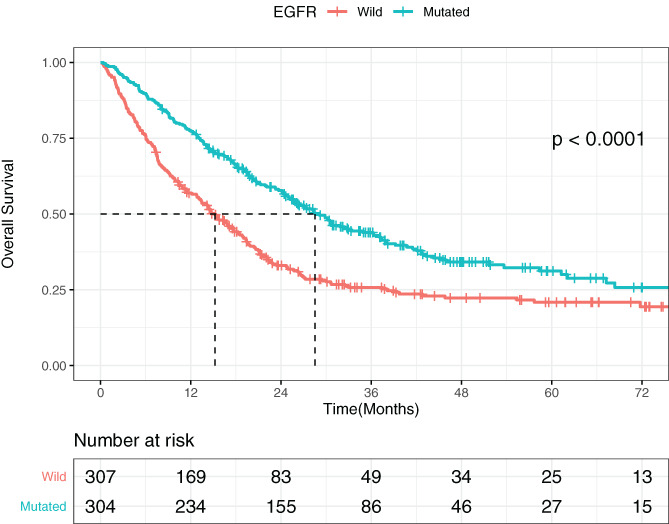
The difference in the overall survival between EGFR‐mutated and wild‐type groups

Almost all NSCLC patients had developed brain metastasis in 5 years. As is demonstrated in Figure 1, EGFR‐mutated patients had significantly better overall survival compared with wild‐type patients (*P* < .0001). The median OS for the EGFR positive NSCLC was significantly greater than the wild‐type cohort.

### 3.3 Prognostic value of the number of brain metastases

Suggested in the univariate analysis, the number of brain metastases had a statistically significant association with OS in both EGFR‐mutated patients (No. of brain metastases = 2: HR = 1.102, 95% CI = 0.680–1.786; No. of brain metastases ≥3: HR = 1.632, 95% CI = 1.164–2.286; *P* = .010) and EGFR wild‐type patients (No. of brain metastases = 2: HR = 1.034, 95% CI = 0.639–1.365; No. of brain metastases ≥3: HR = 1.261, 95% CI = 0.973–1.634; *P* = .039).

**FIGURE 2:**
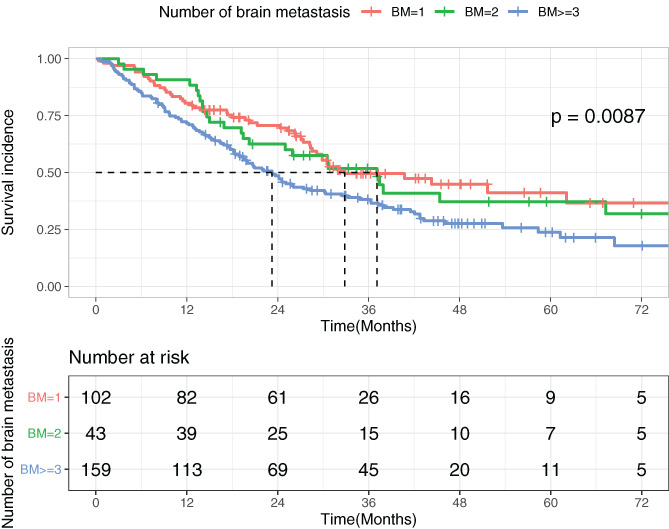
Survival incidence of patients with different numbers of brain metastases in the EGFR‐mutated cohort

**FIGURE 3:**
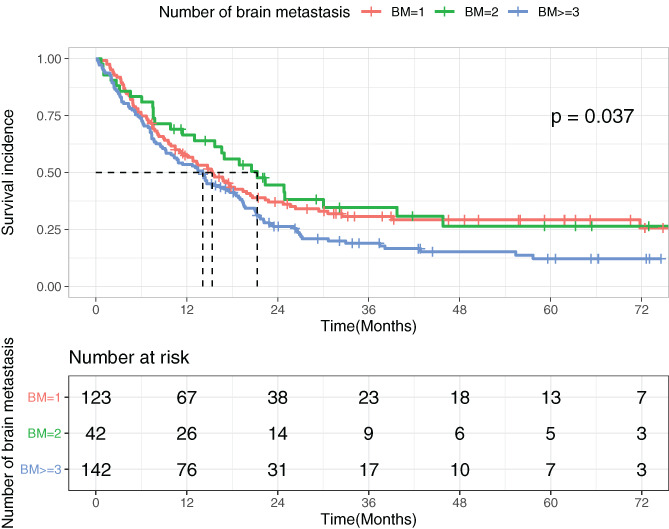
Survival incidence of patients with different numbers of brain metastases in the EGFR wild‐type cohort

We further divided both EGFR‐mutated (*N* = 304) and wild‐type cohorts (*N* = 307) into three subgroups according to the number of brain metastases and viewed the difference in survival incidence among subgroups with one, two, and three or more brain metastases. It turned out that the number of brain metastases had a crucial impact on disease progression (for EGFR‐mutated patients: *P* = .0087; for EGFR wild‐type patients: *P* = .037). Patients with three or more brain metastases had the worst survival incidence regardless of their mutation status (shown in Figures 2 and 3).

### 3.4 Supplementary tables

There were significant differences between the clinical characteristics of 304 patients with EGFR mutations and those of 307 patients without. The characteristics of patients at the time of their initial lung cancer diagnosis are shown in Table S1, while their characteristics at the time of their initial brain metastasis diagnosis can be seen in Table S2.

## 4 DISCUSSION

Lung cancer accounts for 20% malignancy‐related deaths worldwide.^19^ Among the entire patient population, the adenocarcinoma subtype is prevalent.^18^ Nearly 70% NSCLC patients are diagnosed at an advanced stage^17^ and thus have a poor prognosis. The most common metastatic sites include brain, liver, and bones,^20^ consistent with our baseline information (available in Table S1). In NSCLC patients, the incidence of brain metastasis was above 20%, up to nearly 50%.^9^, ^21^, ^22^ Patients included in our study were all harboring brain metastases and there was no statistically significant difference in the number of brain metastases between the EGFR mutation positive cohort and the wild‐type patient cohort when patients were first diagnosed with brain metastasis (*P* = .233). According to our known literature, brain metastasis was more frequent in EGFR‐mutated patients,^23^ especially among never‐smokers.^24^ Our study also vindicated that EGFR‐mutated patients had a notably better prognosis than wild‐type patients. Several studies showed similar results.^12^, ^14^, ^16^

Due to the fact that the number of brain metastases has been found significantly related to the EGFR mutation status,^15^ whether the number of brain metastases is an independent prognostic factor or the impact is due to EGFR mutation is worthy of exploration. A previous study suggested that the number of brain metastases merely impacted outcomes of wild‐type patients,^16^ which was contrary to our result. In our study, the number of brain metastases was found to have a significant association with OS with both groups in the univariate analysis. Although the number of brain metastases was not a statistically independent prognostic factor (*P* = .089) in the EGFR wild‐type cohort in the multivariate analysis, we still believe it might serve as a considerable influential factor accounting for the EGFR mutation status given its impact shown in later illustrations. Figures 2 and 3 show that patients with three or more brain metastases had the worst survival incidence in both EGFR‐mutated and wild‐type groups. The number of brain metastases served as a prognostic factor regardless of their mutation status. In Figure 3, the impacts of extracranial metastases and chemotherapy were excluded, which might have masked the impact of the number of brain metastases on overall survival in the multivariate analysis of wild‐type patients. Furthermore, there were subjects who survived more than 3 years (> 36 months) after brain metastasis among wild‐type cohort. It is interesting to discuss potential treatment that might lead to their better survival, such as immunotherapy or other treatment options.

In the current study, the numbers of patients in EGFR‐mutated group and wild‐type group were nearly equal. This distribution was consistent with the whole Asian NSCLC patients in which approximately 50% cases had EGFR mutations.^25^ As is known, EGFR, related to cell proliferation and survival,^7^ plays an important role in the progression of lung cancer and is easily mutated. It is assumed that the EGFR pathway has a crucial role in the metastasis process of NSCLC.^26^ With an incidence of around 10% of all NSCLC patients,^27^ the EGFR mutation prevalence varies among different populations. For example, in Caucasian NSCLC patients, the proportion of harboring EGFR activating mutation was 10%–20% while around half of Asian patients carried mutations.^9^ And it is more likely for patients with adenocarcinoma to have an EGFR mutation than those with other NSCLC subtypes.^26^ For lung adenocarcinoma patients in East Asia, 40%–60% of them were EGFR mutation positive.^12^

In our study, there was no crucial difference in survival among heterogeneous EGFR mutation subgroups, and the impact of TKI therapy on the survival outcome was not significant for EGFR‐mutated patients, which might result from the limitation of our sample size. The impact of EGFR subtypes and the efficiency of TKIs are to be further defined. As was previously brought up, the specific EGFR mutation subtype might result in different response to targeted therapy. When first‐line treatment fail, the advent of TKIs might provide new opportunities. Mutations in exon 19 and exon 21, which together account for nearly 90% of all EGFR mutations, lead to more sensitive response to TKI therapy agents such as erlotinib, gefitinib, and afatinib.^25^ Further, among the two main subtypes of EGFR mutations, it was previously suggested that treated patients with exon 19 deletions had a longer survival than those with the L858R mutation.^28^ The mechanisms remain to be uncovered.

A summary of representative research reports is shown in Table 2, all of which were published between 2010 and 2020. EGFR mutation status was a risk factor of survival in six studies,^14^, ^23^, ^29^, ^30^, ^31^ including ours. The impact of extracranial metastases was suggested in four studies, the current research also included.^14^, ^30^, ^31^ Performance status was influential to survival outcomes in three studies.^14^, ^17^, ^31^ The role of number of brain metastases was first brought up by Sperduto et al.,^14^ and then further vindicated by Balasubramanian et al.^16^ The current study has been the first to report its significant impact in both EGFR‐mutated and wild‐type patients. Six studies included information regarding EGFR mutation subtypes,^11^, ^23^, ^29^, ^31^, ^32^ in which four studies, ours included,^11^, ^29^, ^31^ reported that no difference existed among different subtypes. But Lee and co‐workers found out that patients with Exon 19 deletion were more sensitive to treatment compared with those with Exon 21 L858 and thus had a better PFS.^32^

**TABLE 2:** A summary of relative research reports

Report	Year	Number of patients	Median age (years)	Ethnicity	Ratio of men to women	Smokers	Adenocarcinoma	Number of patients with EGFR mutations	Exon 19 deletion	Exon 21 mutation	Uneven distribution of EGFR‐mutated patients and wild‐type patients	Difference between EGFR mutation subtypes	Prognostic factors of survival for the whole cohort	Risk factors for EGFR‐mutated patients exclusively	Risk factors for EGFR wild‐type patients exclusively
Current Study	2020	611	56.5	China	1.26	288	502	304	164	117	Sex (*P* < .001), smoking status (P < .001),histology (*P* < .001),metastatic site (*P* = .020)	NO	EGFR mutation status, extracranial metastases condition, chemotherapy adoption condition, the number of brain metastases	Sex	Smoking status, TKI‐targeted therapy
Wang et al^11^	2020	552	≤285	China	1.34	342	501	226	86	79	Brain metastasis	NO	NA	NA	NA
Balasubramanian et al^16^	2020	348	60.5	The United States	0.79	280	NA	91	NA	NA	Smoking status, median number of brain metastases, presence of ECM, symptoms from BM, synchronicity in relation to the diagnosis of primary tumor	NA	KPS	NO	Presence of ECM, number of BM
Lee et al^32^	2018	1312	NA	Australia	NA	NA	NA	1312	751	561	NA	The treatment effect on PFS was greater for exon 19 deletion than exon 21 L858	NA	EGFR‐TKI versus chemotherapy	NA
Sperduto et al^14^	2016	2186	>60	The United States	NA	NA	1521	235	NA	NA	NA	NA	Patient age, KPS, presence of extracranial metastases, number of brain metastases, lack of EGFR and ALK alterations, nonadenocarcinoma	NA	NA
Tomasini et al^23^	2016	142	62	France	1.96	108	127	16	4	2	BM incidence, ethnicity, smoking history, pathology	NA	Mutation status	NA	NA
Hsu et al^29^	2016	543	66	Canada	0.66	NA	NA	121	73	48	The 1‐ and 3‐year cumulative incidence of brain metastases	NO	EGFR status, chemotherapy, EGFR‐TKI, performance status, brain metastasis	NA	NA
Hsiao et al^30^	2013	139	≥60	China	0.7	42	139	89	NA	NA	Sex, smoking	NA	EGFR mutation, ECOG PS, and extracranial metastases	NA	NA
Eichler et al^31^	2010	93	60.9 + 12.0 (mean)	The United States	0.5	53	87	41	13	12	Median time from initial diagnosis to first BM, status of primary tumor, status of extracranial disease, number of brain metastases	NO	EGFR mutation, age, active extracranial disease	NA	NA

Compared with other relative studies, the current study showed a more comprehensive coverage of information, and investigated exclusive risk factors for both EGFR‐mutated and wild‐type patients separately. Our study was among the first few pieces of research focusing on the impact of the number of brain metastases on survival outcomes of NSCLC patients accounting for EGFR mutation status. This research innovatively proposed the significance of the number of brain metastases as a prognostic factor for all patients regardless of their EGFR mutation status. In addition, we also found out that sex was a risk factor exclusively for EGFR‐mutated patients, while smoking status and TKI‐targeted therapy were merely influential among wild‐type patients. Simultaneously, taking the subtypes of EGFR mutations into consideration, we focused on the major mutations of Exon 19 deletion, L858R, G719X, L861Q. And it finally turned out that the specific subtypes of EGFR mutations had no statistically important impact on the overall survival of mutated patients. As studies focusing on Asian populations were relatively lacking, the results of our study were crucial and could offer potential guidance with real‐world evidence to enhance the diagnosis and treatment of Asian NSCLC patients. With follow‐up up to 120 months, the reliability of our results was ensured.

This study had several limitations as follows. First, as a retrospective study, the inherent selection bias was inevitable. To further explore the role of EGFR mutation status in the prognosis of NSCLC patients with brain metastasis, prospective researches are needed. Second, although this research included a large number of NSCLC patients, these patients were all from West China Hospital, Sichuan University. Results from larger‐scale studies in multiple centers should be more convincing. Third, all of the patients included were Asians. Our results need to be validated regarding their relevance to patients in other countries.

As the main cause of cancer‐related deaths, lung cancer has caused numerous deaths in China and throughout the world.^33^ A lot more researches still remain to be done to perfect its diagnosis and treatment in clinical practice.

## 5 CONCLUSION

Our study revealed the potential prognostic role of the number of brain metastases with consideration of the EGFR mutation status, and might help to establish the guidelines of individualizing treatment. It turned out that the number of brain metastases served as a potential prognostic factor in both EGFR‐mutated and wild‐type groups. We also again validated the insight that EGFR‐mutant patients had better prognosis with reliable real‐life evidence.

## CONFLICT OF INTEREST

The authors declare no conflicts of interest.

## AUTHOR CONTRIBUTIONS

All authors had full access to the data in the study and take responsibility for the integrity of the data and the accuracy of the data analysis. *Conceptualization*, W.M.L., C.D.W.; *Methodology*, J.S., Y.X.W.; *Investigation*, J.W.L.; *Formal Analysis*, J.W.L., J.S.; *Resources*, W.M.L., D.L.; *Writing*—*Original Draft*, J.S., C.D.W.,L.J.S.; *Writing*—*Review & Editing*, J.S., C.D.W., L.H.L.; *Visualization*, Q.Y.H., Y.X.W.; *Supervision*, W.M.L.; *Funding Acquisition*, W.M.L.

## ETHICAL STATEMENT

This research was conducted with the approval of the Institutional Review Board in West China Hospital, Sichuan University (NO 2018.420). Informed consent was obtained from all patients included before enrollment, and confidentiality was ensured throughout our research. All authors are accountable for all aspects of the work.

## Supporting information


**Appendix** S1: Supporting Information

## Data Availability

The data that support the findings of this study are available from the corresponding author, Weimin Li, upon reasonable request.
