# Supplementary material for: The number of brain metastases predicts the survival of non‐small cell lung cancer patients with EGFR mutation status
Source: Cancer Rep (Hoboken). 2021 Nov 12;5(9):e1550. doi: 10.1002/cnr2.1550 (PMC9458511; doi:10.1002/cnr2.1550)
Supplement: Supplementary file 1 — Appendix S1: Supporting Information [file CNR2-5-e1550-s001.pdf]

Supplementary Table 1: Patient characteristics at the time of initial lung cancer diagnosis

	EGFR mutated Cohort (N=304)	EGFR wild-type Cohort (N=307)	*P*
**Age, median (range)**	**56(31-81)**	**57(25-83)**	**0.382**
**Age**			
≥60 yr	105	109	0.802
< 60 yr	199	198	
**Sex n(%)**			
Male	122	219	<0.001
Female	182	88	
**Smoking status n(%)**			
Smokers	89	199	<0.001
Non-smokers	215	108	
**CEA level**			
Negative	122	123	0.080
Positive	172	173	
**Histology**			
LUAD	275	227	<0.001
LUSC	16	57	
LASC	8	6	
Others	5	17	
**TNM Stage**			
I	21	21	0.631
II	15	11	
III	50	61	
IV	218	214	
**Metastatic Site**			
Brain	139	162	0.020
Lung	54	38	
Liver	4	9	
Bone	49	28	
Other	58	70	

Abbreviations: EGFR- epidermal growth factor receptor; CEA-carcino-embryonic antigen; LUAD-lung adenocarcinoma; LUSC-lung squamous carcinoma; LASC-lung adenosquamous carcinoma; TNM-Tumor-Node-Metastases.

Supplementary Table 2: Patient characteristics at the time of brain metastasis

	EGFR mutated Cohort (N=304)	EGFR wild-type Cohort (N=307)	*P*
**Age, median (range)**	56.5(32-81)	57(25-83)	0.382
**Age**			
≥60 yr	113	113	0.926
< 60 yr	191	194	
**CEA level**			
Negative	122	123	0.080
Positive	172	173	
**No. of brain metastases** n(%)			
1	102	123	0.233
2	43	42	
≥3	159	142	
**Extracranial metastases (ECM) n (%)**			
YES	124	142	0.173
NO	180	165	
**EGFR Mutated Subtype**			
Exon 19 deletion	164		
L858R	117		
G719X	13		
L861Q	6		
Other	4		
**First-line treatment n(%)**			0.018
SRS±surgery	19	29	
WBRT±surgery	6	5	
SRS+WBRT	20	18	
Chemotherapy	68	79	
**TKI targeted therapy n(%)**			
Yes	266	92	<0.001
No	38	215	

Abbreviations: EGFR- epidermal growth factor receptor; CEA-carcino-embryonic antigen; No. of brain metastases-number of brain metastases; ECM- Extracranial metastases; SRS-stereotactic radiosurgery; WBRT-whole brain radiation therapy; TKI-tyrosine kinase inhibitors.
